# Measuring Health Care Access and Quality to Improve Health in Populations

**Published:** 2010-06-15

**Authors:** Thomas E. Kottke, George J. Isham

**Affiliations:** HealthPartners, Inc; HealthPartners, Inc, Minneapolis, Minnesota

## Abstract

Poor health status, rapidly escalating health care costs, and seemingly little association between investments in health care and health outcomes have prompted a call for a "pay-for-performance" system to improve population health. We suggest that both health plans and clinical service providers measure and report the rates of 5 behaviors: 1) smoking, 2) physical activity, 3) excessive drinking, 4) nutrition, and 5) condom use by sexually active youth. Because preventive services can improve population health, we suggest that health plans and clinical service providers report delivery rates of preventive services. We also suggest that an independent organization report 8 county-level indicators of health care performance: 1) health care expenditures, 2) insurance coverage, 3) rates of unmet medical, dental, and prescription drug needs, 4) preventive services delivery rates, 5) childhood vaccination rates, 6) rates of preventable hospitalizations, 7) an index of affordability, and 8) disparities in access to health care associated with race and income. To support healthy behaviors, access to work site wellness and health promotion programs should be measured. To promote coordinated care, an indicator should be developed for whether a clinical service provider is a member of an accountable care organization. To encourage clinical service providers and health plans to address the social determinants of health, organizational participation in community-benefit initiatives that address the leading social determinants of health should be assessed.

## Background

Poor health status, rapidly escalating health care costs, and seemingly little association between investments in health care and health outcomes have prompted a call for a "pay-for-performance" system to improve population health (1). The goal is to link structure and process to outcomes in the health system, which is the set of institutions and actors that affect people's health, such as organizations that deliver care, health plans, educational systems, and city and county governments. Linking these organizations will contribute to the control of health care costs, improve the health of the US population relative to the health of other developed nations (2), and reduce disparities by region, race, ethnicity, and educational attainment (3).

The lack of tools to measure the effect of clinical services on US population health is rooted in the historical development of the American clinical health care system, which evolved to respond to the acute care needs of the individual: relief of pain and suffering through diagnosis, therapeutic intervention, and reassurance (4). Responsibility for population health needs was with the public health sector alone, and the effect on health of social policies related to education, work, transportation, and other factors was neglected. The Centers for Disease Control and Prevention might be considered the national population health agency, and many state health agencies monitor population health, but these agencies do not have regulatory authority over the health care delivery system. Many local public health agencies are mostly safety net providers. Notions of accountability for population health are underdeveloped at all levels.

Although clinical care accounts for only a small portion of the population health determinants (5), clinical service providers and health plans can contribute to population health initiatives by promoting healthy behaviors and providing clinical preventive services. At a population level, the behaviors that most powerfully affect health are physical inactivity, unhealthy diets, tobacco use, and excessive alcohol consumption (6,7). These behaviors can shorten life expectancy by 10 or more years (8,9). Behavioral support, when delivered with sufficient intensity in settings such as work sites, increases people's odds of adopting and maintaining a healthy lifestyle (10,11). Behavioral and social support is necessary to increase the prevalence of healthy lifestyles because, even when presented with the opportunity to adopt a healthy lifestyle, people still must choose a healthy lifestyle. They are unlikely to do so in a physical and social environment that encourages poor health habits.

Properly selected clinical preventive services also improve population health (12). People are more likely to receive appropriate preventive services when quality assessment systems ensure that they are informed about the benefits of the services and invited to accept the services.

Clinical indicators can identify gaps in access to care — an indicator of quality — and guide the application of incentives to close the gaps. Reporting clinical indicators of population health may also increase the salience of health incentive programs to stakeholders such as clinicians or purchasers of health services, who might be more focused on clinical performance than on long-term mortality trends. The level of clinical indicators can change more rapidly than death rates and longevity, and thus, may give more immediate feedback about the effectiveness of intervention programs. For example, feedback can be provided about positive changes in smoking rates and physical activity rates long before the effect on mortality can be observed.

## Choosing Intervention Strategies to Measure

A list of access and quality indicators related to population health cannot be developed without asking what intervention strategies will improve population health. We believe that 4 clinical care system strategies are strong candidates. The first is to increase rates of healthy behaviors and the delivery of preventive services in traditional settings of health services delivery. The second is to support healthy lifestyles and increase access to health care by extending the clinical setting beyond the doctor's office, for example, by providing wellness and health promotion services in work sites, the dominant social environment in the United States. The third is to develop a system by which clinical care organizations collaborate among themselves to coordinate care and reduce the illnesses and deaths that result from poor communication (13,14). The fourth is to offer clinical service providers and health plans incentives to participate with other sectors in the community (eg, education, transportation, housing, food) to develop policies and programs to improve population health. We describe indicators that are available to promote the first strategy and suggest a set of indicators that could be developed to promote the other strategies.

## Available Indicators

Although nearly 3 dozen indicators are considered current and valid by the Institute of Medicine's Committee on the *State of the USA Health Indicators* (15), nearly all of them are limited in the health domains they assess or the populations they cover. For example, the Healthcare Effectiveness Data and Information Set (HEDIS) indicators, produced by the National Committee for Quality Assurance (NCQA), report the performance of participating health plans (16). In 2008, however, health plans covered only half of Americans, and only half of all health plans reported these indicators. Another organization, the Institute for Healthcare Improvement, has proposed mortality and whole system indicators for health care systems, but these are yet to be implemented. A third example is Minnesota HealthScores, a community-wide program that includes nearly all Minnesota payers. It uses HEDIS and composite indicators of quality of care that bundle many aspects of care performance by condition (17). However, Minnesota HealthScores reports quality of care indicators only for depression, diabetes, and vascular disease, and only for Minnesota. With few exceptions the valid indicators that apply to the entire US population are collected and reported only by the federal government. A notable exception is the Commonwealth Fund, which reports health care system performance and offers international comparisons for some indicators (18).

To inform discussion by policy makers and the public about population health, the State of the USA Project commissioned the Institute of Medicine in 2008 to convene an expert committee to recommend 20 county-level indicators of the health of the United States (15). The committee selected indicators with attention to the availability of data that could be used to report rates at the county level and to make comparisons with other countries. Six indicators of health behavior — smoking, physical activity, excessive drinking, nutrition, obesity, and condom use by sexually active youth in grades 9 through 12 — were selected. Another 6 indicators were selected to characterize the health care systems: health care expenditures; insurance coverage; unmet medical, dental, and prescription drug needs; preventive services; childhood vaccination; and preventable hospitalizations.

We recommend tracking 5 of the 6 health behaviors in a pay-for-population health initiative (Table 1). Although tracking body mass index as an intermediate outcome is useful, obesity is not a behavior per se; therefore, unlike the *State of the USA* report, we have not included it in our list. Regarding the indicators of health care system performance, we recommend tracking the 6 indicators recommended in the *State of the USA* report (15) (Table 2). To draw attention to the economic burden of health care and disparities in access to care, we also recommend 2 indicators that can be calculated from the indicators in the *State of the USA* report and federal data on per capita income, race, and ethnicity.

## Indicators To Be Developed

We suggest that 3 indicators of access and quality be developed for paying health plans or others to improve population health: provision of wellness and health promotion programs, participation in accountable care organizations, and participation in initiatives to benefit communities. Data and, in some cases, methods are not yet available to characterize counties with these indicators.

### Provision of wellness and health promotion programs

The work site is an effective venue to deliver interventions that support healthy behaviors (10), and NCQA now offers accreditation for wellness and health promotion programs (36). Work site wellness and health promotion programs can improve nutrition and physical activity patterns (37). These programs can also reduce tobacco use and hazardous use of alcohol. Approximately 75% of adults aged 20 to 64 are employed (38), and many other adults are a spouse or domestic partner of someone who is employed. In principle, work site wellness and health promotion programs could also offer support in lifestyle skills, such as parenting and financial management, that lie beyond the traditional domain of health care but have a substantial effect on health. Because work site wellness and health promotion programs both reduce health care costs and increase productivity, employers can experience a positive return on investment from sponsoring the programs through increases in productivity alone. Examples of increased productivity include less absenteeism and more productivity.

The indicator that we suggest is the proportion of adults with access to an accredited work site wellness and health promotion program. To assess this rate, an appropriate question could be added to the Current Population Survey (30), the American Community Survey (31), or the US Census Bureau's Surveys of Business Owners (42).

### Participation in accountable care organizations

Poor coordination of care, particularly during transitions between the hospital and the ambulatory care setting, causes avoidable illness and death. This problem can be mitigated through accountable care organizations (ACOs), which are actual or virtual partnerships designed to coordinate care across transitions (13,14). The core of an ACO is effective primary care. For primary care practices to become an ACO, they need at least 8 attributes (14):

- complete and timely information about patients and the services they are receiving.
- technology and skills for population management and coordination of care.
- adequate resources for patient education and self-management support.
- a culture of teamwork among the staff of the practice.
- coordinated relationships with specialists and other providers.
- the ability to measure and report on the quality of care.
- infrastructure and skills for management of financial risk.
- a commitment by the organization's leadership to improve value as a top priority, and a system of operational accountability to drive improved performance.

Although ACOs should be able to improve quality of care, there is not yet evidence that they improve population health. The indicator that we suggest is whether a clinical service provider is a member of an ACO.

### Participation in initiatives to benefit communities

Many of the most powerful determinants of health — for example, transportation, food, employment, social exclusion, and the social gradient — lie outside of the purview of health care (5). Population health could be improved if health care organizations would collaborate with other sectors (eg, housing, transportation, food, economic opportunity) to address these issues. Health plans and hospitals frequently employ substantial numbers of workers in a community. If they are not for profit, they also have obligations to benefit the community.

The Robert Wood Johnson Foundation program Leadership for Healthy Communities (43) is an example of a program that has engaged stakeholders both within and beyond the health care sector to address community characteristics and resources that affect health. The focus of the program is active living and healthy eating to prevent childhood obesity. Minnesota is developing the accountable health communities initiative. The intent of an accountable health community is to bring together health care, schools, work sites, local public health agencies, faith communities, chambers of commerce, nongovernmental agencies, governmental agencies, and others whose policies have an effect on health. The goal is to address social conditions that affect health but lie outside of the health services delivery sector.

The indicator we suggest is organizational participation in community-benefit initiatives that address the leading social determinants of health. NCQA would be an appropriate organization to develop the criteria, and America's Health Insurance Plans would be an appropriate organization to administer the survey.

## Unresolved Issues

If a pay-for-population health initiative is to be implemented, criteria for most of our proposed indicators exist but they must be developed for 3 others. More difficult questions to answer are who would pay for the services and what organizations would be eligible to provide the services. The answer to the latter question is fairly clear for clinical preventive services, but it is less clear for work site wellness and health promotion programs. Would only health plans be eligible, or would any company that offered NCQA-accredited wellness and health promotion services be eligible? Would group purchasing allow small employers to offer wellness and health promotion programs? How would community-benefit initiatives that address the leading social determinants of health be evaluated? How would the contributions of the participating organizations be parsed? Might the agency purchasing population health write a performance contract? These questions can be answered only through an iterative process of negotiation among employers, purveyors of health promotion programs, health plans, communities, and the other stakeholders.

A fundamental requirement for any pay-for-population health initiative is performance data. Ideally, these data would be available to make comparisons at the county level, but the data are not available for some indicators. As part of the pay-for-population health system development, the appropriate federal agencies should be encouraged to collect data that can be reported at the county level.

## Summary

Data are available to measure health care access and quality as reflected by 5 health behavior indicators and 8 health care indicators. Most of the data are collected by federal agencies and are available yearly at the state or county level. Additional indicators that should be developed include whether employees have access to wellness and health promotion services through the work site, whether a health care organization is a member of an ACO, and whether a health plan collaborates in community-benefit initiatives that address the leading social determinants of health. Data for these indicators should be collected at the county level by appropriate federal agencies.

## Figures and Tables

**Table 1:** Health Behaviors That Are Measurable Indicators of Health Care Access and Quality

**Behavior and Definition^a^**	**Data Source**
**Smoking:** Percentage of adults who have smoked ≥100 cigarettes in their lifetime and who currently smoke some days or every day.	NHIS (19), BRFSS (20), and WHO (21)
**Physical activity:** Percentage of adults meeting the recommendation for moderate physical activity (at least 5 days per week for 30 minutes per day of moderate-intensity activity or at least 3 days per week for 20 minutes per day of vigorous-intensity activity).	NHIS (19) and BRFSS (20)^b^
**Excessive drinking:** Percentage of adults consuming 4 (women) or 5 (men) or more drinks on 1 occasion and/or consuming more than an average of 1 (women) or 2 (men) drinks per day during the past 30 days.	NHIS (19), BRFSS (20), and WHO (23)
**Nutrition:** Percentage of adults with a good diet (conformance to federal dietary guidance) as indicated by a score of ≥80 on the Healthy Eating Index (24).	NHANES (25)^c^
**Condom use:** Proportion of youth in grades 9-12 who are sexually active and do not use condoms, placing them at risk for sexually transmitted infections.	YRBSS (26) and WHO (27)^d^

Abbreviations: NHIS, National Health Interview Survey; BRFSS, Behavioral Risk Factor Surveillance System; WHO, World Health Organization; NHANES, National Health and Nutrition Examination Survey; YRBSS, Youth Risk Behavior Surveillance System.

a Detailed information regarding each indicator is available in the Institute of Medicine's report *State of the USA Health Indicators: Letter Report* (15).

b WHO has implemented a global strategy on diet, physical activity, and health (22), but data are not yet available for international comparisons.

c The Healthy Eating Index is not well-suited for global comparisons, and uniform data for global comparisons are not available.

d WHO collects data on condom use among people aged 15-24 years, so the data are not strictly comparable.

**Table 2:** Health Care Sector Attributes That Are Measurable Indicators of Health Care Access and Quality

**Attribute and Definition^a^**	Data Source
**Health care expenditures:** Per capita health care expenditures.	NHEA (28 and OECD (29)
**Insurance coverage:** Percentage of adults without health care coverage through insurance or entitlement.	CPS (30) and ACS (31)
**Unmet medical, dental, and prescription drug needs:** Percentage of noninstitutionalized people who did not receive or delayed receiving needed medical services, dental services, or prescription drugs during the previous year.	MEPS (32)
**Preventive services:** Percentage of adults who are up to date with age-appropriate screening services^b^ and influenza vaccination.	MEPS (32)
**Childhood vaccination:** Percentage of children aged 19-35 months who are up to date with recommended vaccinations.^c^	NIS (33)
**Preventable hospitalizations:** Hospitalization rate for ambulatory-care-sensitive conditions.^d^	PQI (34)
**Index of affordability:** Per capita health expenditures as a percentage of per capita income.	NHEA (28) and BEA (35)
**Disparities in access to health care:** Percentage of (noninstitutionalized) poor who did not receive or delayed receiving needed medical services, dental services, or prescription drugs during the previous year divided by the percentage of nonpoor reporting the same barrier. Data also presented for racial/ethnic minorities divided by data for non-Hispanic whites.	MEPS (32)

Abbreviations: NHEA, National Health Expenditure Account; OECD, Organisation for Economic Co-operation and Development; CPS, Current Population Survey; ACS, American Community Survey; MEPS, Medical Expenditure Panel Survey; NIS, National Immunization Survey; PQI, Agency for Healthcare Research and Quality Prevention Quality Indicators; BEA, Bureau of Economic Analysis.

a The Institute of Medicine's report *State of the USA Health Indicators: Letter Report* (15) has detailed information regarding health care expenditures; insurance coverage; unmet medical, dental, and prescription drug needs; preventive services; childhood vaccination; and preventable hospitalizations.

b Blood pressure check within the previous 2 years; cholesterol check within the previous 5 years; fecal occult blood test within the previous 2 years; ever having had colonoscopy or sigmoidoscopy; influenza vaccination within the previous year; and Papanicolaou test within the previous 3 years and mammogram within the previous 2 years as appropriate for sex and age group.

c The recommended series consists of 4 doses of diphtheria and tetanus toxoids and pertussis vaccine; 3 doses of polio vaccine; 1 or more doses of measles, mumps, and rubella vaccine; 3 doses of *Haemophilus influenzae* type b vaccine; 3 doses of hepatitis B vaccine; and 1 or more doses of varicella (chickenpox) vaccine.

d Short-term and long-term complications of diabetes, uncontrolled diabetes, lower-extremity amputations among patients with diabetes, perforated appendicitis, chronic obstructive lung disease, congestive heart failure, angina without a procedure, hypertension, low birth weight, dehydration, bacterial pneumonia, urinary tract infections, and adult asthma.
